# The effectiveness of the 13-valent pneumococcal conjugate vaccine against hypoxic pneumonia in children in Lao People's Democratic Republic: An observational hospital-based test-negative study

**DOI:** 10.1016/j.lanwpc.2020.100014

**Published:** 2020-09-06

**Authors:** Rupert Weaver, Cattram D. Nguyen, Jocelyn Chan, Keoudomphone Vilivong, Jana Y.R. Lai, Ruth Lim, Catherine Satzke, Malisa Vongsakid, Paul N. Newton, Kim Mulholland, Amy Gray, Audrey Dubot-Pérès, David A.B. Dance, Fiona M. Russell

**Affiliations:** a Centre for International Child Health, Department of Paediatrics (WHO Collaborating Centre for Child and Neonatal Health Research and Training), The University of Melbourne, Melbourne, Victoria, Australia; b Department of Paediatrics, The University of Melbourne, Melbourne, Australia; c Murdoch Children's Research Institute, Melbourne, Australia; d Lao-Oxford-Mahosot Hospital-Wellcome Trust Research Unit, Mahosot Hospital, Vientiane, Lao PDR; e Australian National University, Canberra, Australia; f Department of Microbiology and Immunology at the Peter Doherty Institute for Infection and Immunity, Melbourne, Australia; g Centre for Tropical Medicine & Global Health, University of Oxford, Oxford, UK; h London School of Hygiene and Tropical Medicine, London, UK; i Unite des Virus Emergents (UVE: Aix-Marseille Univ – IRD 190 – Inserm 1207 – IHU Mediterranee Infection), Marseille, France

**Keywords:** Pneumococcal conjugate vaccine, Pneumonia, Hypoxia, Vaccine effectiveness, Children, Test-negative

## Abstract

**Background:**

Pneumococcal pneumonia is a leading cause of childhood mortality. Pneumococcal conjugate vaccines (PCVs) have been shown to reduce hypoxic pneumonia in children. However, there are no studies from Asia examining the effectiveness of PCVs on hypoxic pneumonia. We describe a novel approach to determine the effectiveness of the 13-valent PCV (PCV13) against hypoxia in children admitted with pneumonia in the Lao People's Democratic Republic.

**Methods:**

A prospective hospital-based, test-negative observational study of children aged up to 59 months admitted with pneumonia to a single tertiary hospital in Vientiane was undertaken over 54 months. Pneumonia was defined using the 2013 WHO definition. Hypoxia was defined as oxygen saturation <90% in room air or requiring oxygen supplementation during hospitalisation. Test-negative cases and controls were children with hypoxic and non-hypoxic pneumonia, respectively. PCV13 status was determined by written record. Vaccine effectiveness was calculated using logistic regression. Propensity score and multiple imputation analyses were used to handle confounding and missing data.

**Findings:**

There were 826 children admitted with pneumonia, 285 had hypoxic pneumonia and 377 were PCV13-vaccinated. The unadjusted, propensity-score adjusted and multiple-imputation adjusted estimates of vaccine effectiveness against hypoxic pneumonia were 23% (95% confidence interval: -9, 46%; p=0•14); 37% (6, 57%; p=0•02) and 35% (7, 55%; p=0•02) respectively.

**Interpretation:**

PCV13 is effective against hypoxic pneumonia in Asia, and should be prioritised for inclusion in national immunisation programs. This single hospital-based, test-negative approach can be used to assess vaccine effectiveness in other similar settings.

**Funding:**

Funded by the Bill & Melinda Gates Foundation.

**Research in context**

**Evidence before this study**

We searched Medline (Ovid), Embase (Ovid) and Pubmed for reports published before January 2019 for terms: pneumococcal conjugate vaccine, pneumonia, pneumo*, *Streptococcus pneumoniae*. We searched for studies that evaluated the vaccine efficacy and vaccine effectiveness (VE) of the 13-valent pneumococcal conjugate vaccine (PCV13) against outcomes of hypoxic pneumonia and mortality attributable to pneumonia. The population of interest was children under five years of age, using either randomised control trials (RCTs), observational population-based or case-control studies. We identified six studies assessing PCV VE against pneumonia mortality. This included four observational studies that reported a PCV VE between 8-71% against pneumonia-related mortality. Additionally, one RCT was identified, which reported a 54% reduction in mortality attributable to pneumonia (p=0•73). We identified two observational studies that found a 47-61% reduction in hypoxic pneumonia post PCV13 introduction. All studies were in Europe, Africa and South America. There are no published studies on the PCV13 VE against pneumonia or hypoxic pneumonia in Asia, and no studies using a single hospital-based approach.

**Added value of this study**

We conducted a prospective observational study in a single hospital to determine the PCV13 VE against hypoxic pneumonia and pneumonia requiring supplementary oxygen in children compared to non-hypoxic children with pneumonia in the Lao People's Democratic Republic (Lao PDR, Laos) between December 2013 and June 2018. We found that PCV13 reduced hypoxic pneumonia and pneumonia requiring supplementary oxygen by 37% ( 95% confidence interval: 6, 57%; p=0•02) in children with pneumonia. This is the first study documenting the impact of PCV13 on hypoxic pneumonia in Asia. We outline a novel method using a test-negative design to determine the VE using a single hospital approach.

**Implications of all the available evidence**

The WHO recommends that evaluation of the impact of PCVs on pneumococcal diseases should be undertaken to inform vaccine policy decisions. However, in LMICs, population-level surveillance data may not be available, and undertaking large-scale epidemiological studies is expensive and time consuming. In these settings, case-control studies may be utilised to measure VE. However, case-control studies may be vulnerable to confounding and other challenges in the selection of appropriate controls. Previously, Madhi et al noted in their case-control study evaluating the VE of PCV7 and PCV13 against presumed bacterial pneumonia, confirmed radiologically, that hospital controls were more comparable to cases than community controls in regards to demographic data. In our study, we outline a novel method using a test-negative design that can be conducted within a single hospital. This is a simple methodological approach, which can be utilised in other settings. Our study is the first to report on the VE of PCV13 in Asia. Asian countries have been very slow to introduce PCV into their national immunisation programs. These results provide supportive evidence for decision-makers in other countries, particularly LMICs, to include PCV into their national programs. PCV13 is particularly effective against the most severe form of pneumonia (hypoxic pneumonia), which is consistent with other studies. As hypoxia is the precursor to mortality, our results suggest that PCV13 will contribute to a reduction in childhood mortality in Laos and more broadly in the region, providing evidence of impact to support the continued use of PCV13 in Laos.

Alt-text: Unlabelled box

## 1 Introduction

Globally, lower respiratory infections, including pneumonia, are a leading cause of morbidity and mortality in children under five years old, causing 652,572 deaths annually [1], predominantly in low- and middle-income countries (LMICs) [2]. *Streptococcus pneumoniae* (pneumococcus) is estimated to cause over half of all pneumonia-related deaths in children under five years old [1]. There are more than 90 different pneumococcal serotypes, but knowledge is limited of their relative contribution to disease and their geographic and demographic variation [3].

This high burden of disease prompted the development of the infant pneumococcal conjugate vaccines (PCVs), including the 13-valent PCV (PCV13), which covers 13 of the most common disease-causing pneumococcal serotypes. In randomised controlled trials (RCTs), the efficacy of PCV against radiologically-confirmed childhood pneumonia ranges from 20-37% [4,5]. One RCT found a 16% (95% confidence interval [CI]: 3, 28%) reduction of all-cause mortality in children under two years old [4]. Observational studies have found an 8-71% reduction in pneumonia-related mortality in children aged under two years old following the introduction of PCV [6], [7], [8]. One study noted a greater decline in pneumonia-related mortality in lower socioeconomic groups (10% v 24%) [8]. No RCTs have assessed vaccine efficacy against hypoxic pneumonia, a precursor of mortality. There are only two observational studies from Africa, which reported 47% and 61% reductions in hypoxic pneumonia in children under five years old following PCV13 introduction [9,10]. No studies have assessed the impact of the currently available PCVs against any pneumonia endpoint in Asian LMICs [11,12].

As PCVs are expensive, governments require evidence of their health benefits within their populations. However, measuring the impact or effectiveness of PCVs is challenging. To document PCV impact on pneumonia, countries often rely on routinely collected administrative data on hospitalisations, which may not be complete, as well as population denominators, which are often not available. Vaccine effectiveness (VE) is commonly measured using cohort or case-control studies. Cohort studies require follow-up of vaccinated and unvaccinated participants for the occurrence of study endpoints, which can be expensive and difficult to implement in low-resource settings [13]. Measuring VE using the standard case-control design is challenging due to difficulties in selecting controls that are representative of the source population of the cases [13]. In particular, biases can arise due to differences in health-seeking behaviour of the cases and controls [14]. Test-negative studies, a variant of case-control studies, have been proposed as an alternative approach for estimating VE [14]. With the test-negative design, cases and controls present with the same clinical syndrome, but differ with respect to their test result for a disease or pathogen of interest [15]. Because the cases and controls present to health facilities with similar signs and symptoms, they are likely to come from the same catchment areas and have comparable health-seeking behaviour and data quality [15]. Test-negative studies can also provide practical advantages as they can be carried out within a single hospital or surveillance system [13]. Similar approaches (e.g. “Broome method” [16], “indirect cohort” method [17]) have been used to examine the effectiveness of PCVs against vaccine-type invasive pneumococcal disease in settings where diagnostic testing is available.

Pneumonia is a leading cause of infant mortality in the Lao People's Democratic Republic (Laos) [18]. Universal health care did not exist in Laos until recently, and supplementary oxygen was prohibitively expensive for families [19]. As such, the PCV13 has great potential to alleviate this burden of disease on the most vulnerable. In October 2013, Laos introduced PCV13 into the national childhood vaccination program, supported by Gavi, the Vaccine Alliance. As the country is undergoing Gavi transition, the Ministry of Health requested evidence of the health benefits of PCV to support its ongoing use. The vaccine is administered in a “3+0” schedule at six, ten, and 14 weeks of age, and during the initial roll-out, catch-up vaccination was offered to infants up to 12 months old. In this study, we aimed to determine the effectiveness of PCV13 on hypoxic pneumonia in children in Laos, using a prospective single-hospital approach. We use a novel approach that enrols all children with pneumonia, and defines “test-negative” cases and controls as hypoxic and non-hypoxic pneumonia patients, respectively. This is a modified approach to the “test-negative” design, which usually defines case and control status based upon test results for a pathogen [14].

## 2 Methods

### 2.1 Study site

This study was conducted at Mahosot Hospital in Vientiane, the capital of Laos. It is a 365-bed general tertiary referral hospital and primary care provider to the local catchment area. Approximately 400 children less than five years old are admitted with acute respiratory infections (ARIs) each year. Respiratory infection admissions are known to peak in the hot rainy season in Laos. Oxygen supplementation was available for this study at no cost to the patient and their families. In 2016, the national coverage of the third dose of PCV13 was estimated to be 78% [20].

### 2.2 Study design

This was a prospective hospital-based study of children admitted with an ARI to any paediatric or intensive care ward at Mahosot Hospital, nested within ongoing hospitalised ARI surveillance [21]. The test-negative analysis included pneumonia cases enrolled from December 2013 to July 2018 (when the planned sample size was attained, see “Sample size calculation” section). Six cases (n=1 hypoxic, n=4 not hypoxic, n=1 unrecorded oxygen saturation) enrolled in December 2013 were classified as 2014 cases for analyses using year of enrolment.

### 2.3 Study participants

Children were enrolled into the ARI surveillance if they were aged 0-59 months and admitted with a primary diagnosis of ARI defined as: ≤14-day history of fever or documented fever (>38•0°C, axillary, rectal or oral), and one or more of: cough; rhinitis; dyspnoea or abnormal auscultatory findings (reduced breath sounds or inspiratory crepitations). Only children who fulfilled the definition of pneumonia, i.e. children with cough or difficulty breathing, and one of either tachypnoea (≥60 breaths per minute (bpm) <2 months old; ≥50 bpm if 2-11 months old; or ≥40 bpm if 12-59 months old) or chest indrawing, were included [22]. Children were excluded if their parents did not consent or they did not fit the clinical definition of pneumonia.

### 2.4 Study procedures

Study staff screened potential recruits from Monday to Friday each week. For those eligible, following written, informed consent from parents/guardians, information on demographics, medical history and clinical details were collected through parental interview and review of the medical records, and recorded on a data collection form by study staff. The data collected included: date of enrolment; season of enrolment (Wet (May to September) or Dry (October to April)); number of other people living in the household; source of cooking fuel (electricity, coal, wood or gas); whether the household had piped water; ethnicity of the family; residence (within Vientiane Capital or other provinces); comorbidities (concurrent infection, malnutrition, congenital heart disease, chronic lung disease, cancer, asthma, diabetes, prematurity or low birth weight); required assisted ventilation (continuous positive airway pressure (CPAP) or mechanical ventilation); intensive care admission for >24 hours; outcome (death, discharged alive); and attendance at day-care centre. Each participant had a nasopharyngeal swab taken, which was tested for pneumococcus and human respiratory syncytial virus (HRSV) (See Supplementary methods for details). The pneumococcal and HRSV results were used as a control condition and confounder in this study, respectively. PCV13 vaccination status was recorded from the parent-held immunisation record or health centre immunisation records.

### 2.5 Study measures

The primary outcome was hypoxia, defined as an oxygen saturation of <90% in room air on admission or requiring oxygen supplementation during admission. Oxygen saturation was recorded using the non-invasive Masimo® and Lifebox® oximeters. For the test-negative design, cases were defined as children who met the case definition for pneumonia and were hypoxic. Because oxygen saturations were only recorded on admission, we additionally considered participants to be cases if they required oxygen supplementation during their hospitalisation. Test-negative controls were children with non-hypoxic pneumonia. Children were considered PCV13-vaccinated if they had received at least two doses of PCV13 and were aged between 0-11 months; and for those children older than 12 months, if they had received at least one dose of PCV13 [23]. Children were considered undervaccinated if they had received fewer doses by age group.

### 2.6 Data management

Data collection forms of all participants were reviewed and uploaded into a REDCap database hosted at the Murdoch Children's Research Institute [24,25]. Data were monitored, corrected then double-entered.

### 2.7 Statistical analysis

Categorical data were summarised as numbers and percentages. Continuous data were summarised as medians and interquartile ranges. Age was reported as both a continuous and categorical variable.

Logistic regression was used to estimate the odds ratios (OR) and associated 95% CI of hypoxic pneumonia by PCV13 vaccination status. The ORs were converted to measures of VE using the formula: VE=(1-OR)*100. To handle potential confounding, we used inverse probability of treatment weighting (IPTW), which weights individuals by the inverse of the probability of PCV13 vaccination conditional on covariates (i.e. the propensity score (PS)). The PS was estimated using a logistic regression model with PCV13 status as the outcome variable and potential confounders as covariates. The following variables were included in the propensity score model based on prior subject matter knowledge: age, sex, season, day care attendance, number of other people in the household, comorbidities, date of enrolment, and HRSV infection status. A number of additional variables (maternal education, access to piped water, cooking fuel, ethnicity, and residence in a rural or urban setting) were included in the model based on observed differences between the vaccinated and undervaccinated groups. To assess balance of covariates between vaccinated and undervaccinated groups, we calculated standardised differences for each covariate before and after weighting. Covariates were considered balanced if the weighted standardised differences were <10% [26]. We performed an additional analysis using similar methods to estimate PCV13 VE against a control condition of total pneumococcal carriage (defined as the carriage of any pneumococcal serotype, as measured by the analysis of nasopharyngeal swabs as described in the Supplementary methods). Total pneumococcal carriage was selected as a control condition, as we do not expect PCV13 to affect overall pneumococcal carriage rates [27].

Some children had unknown PCV13 vaccination status and unrecorded oxygen saturation levels. To assess for systematic differences between children with and without missing values, we compared them on demographic and medical characteristics and assessed differences using Chi-squared tests for categorical variables and Wilcoxon rank-sum tests for continuous variables. Primary VE analyses included participants with completely observed data on all variables in the adjusted and unadjusted analyses (i.e. complete case analyses). We examined the sensitivity of the results to the handling of missing data by using multiple imputation (See Supplementary methods). The missing data were imputed using multiple imputation by chain equations (MICE) using the *mi impute chained* command in Stata, with 40 imputed datasets created. The unadjusted and PS analyses were performed separately on each imputed dataset, and the results combined using Rubin's rules [28]. Data cleaning and analysis were conducted using Stata version 15 [29].

### 2.8 Sample size calculation

It was estimated that the PCV13 VE against hypoxic pneumonia was 40% based upon a published study [10]. Assuming 60% of controls were vaccinated and a 2:1 ratio of controls to cases, a sample size of 256 cases and 511 controls would have 90% power to detect an odds ratio of 0.6 (i.e. VE of 40%).

### 2.9 Ethics approval

This study was conducted according to the study protocol approved by the Oxford Tropical Research Ethics Committee (OxTREC reference:1050-13), Laos Ministry of Health National Ethics Committee for Health Research (No 061NECHR), the Ethics Review Committee of the WHO Regional Office for the Western Pacific Region (WPRO-ERC) (reference ID:2013.30.LAO.2.EPI) and The Royal Children's Hospital Human Resources Ethics Committee (HREC) (reference number:33177B).

### 2.10 Role of the funding source

This work was supported by the Bill & Melinda Gates Foundation (OPP1115490) with additional support from the Wellcome Trust. The funders had no role in the study design, data collection, data analysis, data interpretation, or writing of the report. The corresponding author had full access to all data in the study and had final responsibility for the decision to submit for publication.

## 3 Results

**Fig. 1:**
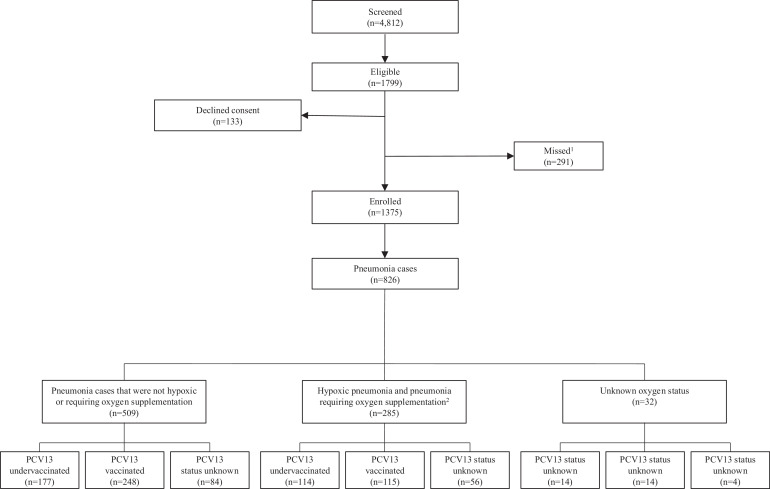
Flow chart of study recruitment of children of children with pneumonia between the age of 0-59 months at the Mahosot Hospital, Vientiane, Lao PDR between December 2013 and July 2018 (n=4,312). Legend: ^1^Missed cases refer to cases not enrolled due to admission and discharge over the weekend.^2^Hypoxic pneumonia and pneumonia requiring oxygen supplementation includes any cases with hypoxic oxygen saturation levels (<90% O_2_) measured (at any stage during admission) and those cases receiving oxygen supplementation at any stage during admission.

Between December 2013 to July 2018, 4,812 children were screened (Fig. 1). There were 1,375 children enrolled in the study for whom consent was given. Of these, 826 children had pneumonia and 96•1% had oxygen saturation recorded. PCV13 vaccination status was confirmed by written record for 82•6% of children with pneumonia.

The median age of the participants with pneumonia (n=826) was 11 months (IQR:4•6-19•9) and 52.1% were infants (under 12 months) (Table 1). Of the children with pneumonia, 55.3% were PCV13-vaccinated, 35.9% were hypoxic, 31•0% received supplementary oxygen and 33•5% were HRSV positive. In total, 11•7% of children had a comorbidity. Of the 285 participants who were classified as cases, 139 (49%) had an oxygen saturation of <90% in room air on admission, and 146 (51%) were not hypoxic on admission, but required supplementary oxygen during their hospitalisation. There were only minor differences between these two groups; those who were hypoxic on admission were less likely to reside in homes that used wood for cooking (43% v 56%), less likely to be admitted during the wet season (35% v 49%), and more likely to be enrolled earlier in the study (e.g. 53% v 39% enrolled before 2016) compared to those who were non-hypoxic on admission but required supplementary oxygen (Supplementary Table 1).

**Table 1:** Characteristics of children admitted with pneumonia, by hypoxic pneumonia v non-hypoxic pneumonia status (n=826).

Characteristics	Total (n=826)	Hypoxic pneumonia (n=285)	Non-hypoxic pneumonia (n=509)	p-value
**Age in months, median (IQR)**	11.4 (4•6-19•9)	8•0 (2•8-15•9)	13•2 (5•7-21•4)	<0•001
**Age <12 months, n (%)**	430 (52.1)	183 (64•2)	236 (46•4)	<0•001
**Female, n (%)**	349 (42•3)	125 (43.9)	211 (41.5)	0•51
**Ethnicity (Lao Loum), n (%)**	717 (86•8)	238 (83•5)	451 (88•6)	0•042
**PCV13 status**^1^**, n/N (%)**				
**Vaccinated**	377/682 (55.3)	115/229 (50.2)	248/425 (58•4)	0•046
**Undervaccinated**	305/682 (44.7)	114/229 (49•8)	177/425 (41•7)	
**Comorbidities**^2^**, n (%)**	97 (11•7)	53 (18•6)	43 (8•4)	<0•001
**HRSV positive, n/N (%)**	257/767 (33•5)	83/261 (31•8)	168/474 (35•4)	0•32
**Urban residence (Vientiane capital district), n (%)**	655 (79•3)	203 (71•2)	428 (84•1)	<0•001
**Daycare attendance**^3^**, n/N (%)**	105/822 (12•8)	20/284 (7•0)	81/507 (16•0)	<0•001
**Mother's level of education higher than junior high school, n/N (%)**	564/733 (76•9)	183/250 (73•2)	358/451 (79•4)	0•06
**Wet season**^4^**, n (%)**	373 (45•2)	121 (42•5)	240 (47•2)	0•20
**Year of enrolment**^5^**, n (%)**				
**2014**	254 (30•7)	76 (26•7)	165 (32•4)	0•005
**2015**	198 (24•0)	55 (19•3)	128 (25•1)	
**2016**	185 (22•4)	83 (29•1)	99 (19•4)	
**2017**	144 (17•4)	50 (17•5)	94 (18•5)	
**2018**	45 (5•4)	21 (7•4)	23 (4•5)	
**Number of other people in household, median (IQR)**	5 (4-7)	5 (4-7)	5 (4-7)	0•41
**Piped water source**^6^**, n (%)**	431 (52•2)	125 (43•9)	289 (56•8)	<0•001
**Wood used for cooking**^7^**, n (%)**	345 (41•8)	142 (49•8)	192 (37•7)	<0•001
**Supplementary oxygen used, n (%)**	256 (31.0)	256 (89.8)	0 (0)	<0.001
**Assisted ventilation**^8^**, n/N (%)**	26/824 (3.2)	25/284 (8.8)	1/508 (0.2)	<0.001
**Wheeze, n/N (%)**	297/661 (44.9)	102/234 (43.6)	195/427 (45.7)	0.61
**Death, n (%)**	9 (1.1)	9 (3.1)	0 (0)	<0.001

Legend: IQR: interquartile range. For categorical variables, data are presented as frequencies (n), or fractions (n/N) for variables with missing data.

1 PCV 13 status: 0-11 months 2 doses of PCV13; >12 months 1 dose; undervaccinated: ≤11 months 0-1 dose; ≥12 months 0 doses of PCV13.

2 Comorbidities included: concurrent infection, malnutrition, congenital heart disease, chronic lung disease, cancer, asthma, diabetes, prematurity or low birth weight.

3 Attendance at daycare indicates children attended daycare for any length of time.

4 Season: wet season refers to period between May to September, dry season refers to period between October to April.

5 Year of enrolment refers to enrolment in study from 1^st^ of January to 31^st^ of December of that year (2013 was merged with 2014 due to low numbers), except 2017 which was January to June.

6 Piped water source: Household had piped water supply.

7 Wood used for cooking: Household had wood as primary cooking fuel as opposed to other sources.

8 Assisted ventilation: CPAP or mechanical ventilation.

Characteristics of children with and without hypoxic pneumonia are shown in Table 1. Children with hypoxic pneumonia were younger (median age: 8 months v 13 months), were less likely to come from homes with piped water (44% v 57%), were less likely to live in Vientiane Capital (71% v 84%), were less likely to attend daycare (7% v 16%) and were less likely to be of Lao Loum ethnicity (84% v 89%) compared to non-hypoxic children (Table 1). Children with hypoxic pneumonia were also more likely to reside in homes that used wood for cooking (50% v 38%) and more likely to have a comorbidity (19% v 8%).

Of the 826 pneumonia patients, 144 (17•4%) had missing PCV13 vaccination status, 36 (4•4%) had unrecorded oxygen saturation levels and 570 (69%) had complete data for all variables in the propensity score analysis. Compared to children with known PCV13 status, those with unknown PCV13 status were enrolled later in the study (e.g. 60% v 42% enrolled after 2015), more likely to reside in homes that used wood for cooking (55% v 39%), less likely to live in a home with piped water (40% v 55%,), less likely to live in Vientiane Capital (62% v 83%) and less likely to be of Lao Loum ethnicity (77% v 89%) (Supplementary Table 2). Children with unrecorded oxygen saturations were enrolled earlier in the study compared to those with recorded levels (e.g. 86% vs 53% enrolled before 2016) (Supplementary Table 3).

There were also differences between vaccinated and undervaccinated children (Supplementary Table 4). Among participants with completely observed data (n=570), undervaccinated children were younger (median age: 6 months v 13 months) and enrolled earlier in the study (e.g. 51% v 24% enrolled before 2015) compared to vaccinated children. Vaccinated children were more likely to come from homes with maternal education levels higher than primary (84% v 73%) and homes with piped water (63% v 43%). Following PS weighting, standardised differences between covariates were <10%, indicating that covariates were balanced between exposure groups (Supplementary Table 4).

The unadjusted VE against hypoxic pneumonia was 23% (95% CI: -9, 46%; p=0•14) (Table 2). When adjusted for confounders using PS analysis the VE was 37% (95% CI: 6, 57%; p=0•02) (Table 2). The unadjusted and PS-adjusted VE using multiple imputation were: 31% (95% CI: 6, 50%; p=0•02) and 35% (95% CI: 7, 55%; p=0•02) respectively (Table 2). The VE against total pneumococcal carriage was -6% (95% CI: -54, 28%; p=0.78) in the PS-adjusted analysis of the control condition (Supplementary Table 5).

**Table 2:** Unadjusted and propensity score adjusted PCV13 vaccine effectiveness (VE) against hypoxic pneumonia^1^. Results are shown for complete case analyses (n=570) and multiple imputation analyses (n=826).

	Unadjusted PCV13 VE (95% CI)	p-value	Adjusted PCV13 VE (PS^2^) (95% CI)	p-value
**Complete case analysis**	23 (-9, 46)	0•14	37 (6, 57)	0•02
**Multiple imputation**	31 (6, 50)	0•02	35 (7, 55)	0•02

Legend:

1 Hypoxic pneumonia (Oxygen saturation (1st measured) either at or during admission: non-hypoxic: ≥90% O_2_ and hypoxic <90%) and cases requiring oxygen supplementation therapy at any stage during admission.

2 PS (Propensity score): individuals were weighted by the inverse probability of the conditional likelihood of being vaccinated using logistic regression on covariates: age, sex, season, day care attendance, number of adults in the household, maternal education, access to piped water, cooking fuel, ethnicity, date of enrolment, residing in a rural or urban setting, comorbidities and HRSV infection status.

## 4 Discussion

Using a novel single hospital-based test-negative study design, this is the first study to show that PCV13 is effective against hypoxic pneumonia in Asian children. Considering the high burden of pneumonia and limited access to treatment in these settings, PCV13 is likely to substantially reduce childhood mortality. Most countries in Asia do not include PCV in their national immunisation programs. Our findings suggest that PCV should be considered a priority for introduction in Asia, especially for children most at risk of pneumonia and in countries with high child mortality.

Our results are consistent with two other observational studies of PCV13 from Africa, which both found reductions in hypoxic pneumonia in children [9,10]. In Malawi, there was a 47% (95% CI: 5%, 70%, p = 0.031) reduction in hypoxic pneumonia post-PCV13 introduction [9]. In The Gambia, there was a 61% (95% CI: 52%, 68%) reduction in the incidence of hypoxic pneumonia post-PCV13 introduction [10]. Our study in Laos found a 37% reduction in hypoxic pneumonia (when adjusted for confounders). Differences in reported effect sizes may be due to a variety of factors, including geographic variation in pneumococcal serotypes that cause pneumonia. There were numerous differences in the study designs: the Malawi study used a time series analysis of pneumonia surveillance data, and the Gambian study compared incidences of hypoxic pneumonia in pre- and post-PCV13 periods using surveillance data, while our study used a PS analysis to estimate VE using cases enrolled in surveillance at a single hospital. All studies included children under the age of five years. However, the Gambian study limited their study to children over the age of two months. The study populations likely differed in demographic factors, comorbidities and other risk factors which may influence the severity of illness or health seeking behaviour [2]. All three studies investigated a “3+0” vaccination schedule with catch up. However, The Gambia achieved higher PCV13 population coverage (96%) compared with Malawi (76%) and our site (78%) [9,10,20]. Although it is unknown what coverage is required to confer herd protection [30], the higher coverage in The Gambia along with longer follow up time may have resulted in greater herd protection and thereby higher vaccine impact. The advantage of the observational vaccine impact study design is that it may capture these indirect effects [31].

No RCTs have used hypoxic pneumonia or mortality due to pneumonia as a primary outcome for PCV vaccine efficacy. However, a meta-analysis of RCTs reported smaller, but comparable, PCV effect sizes against all-cause mortality (11%; [95% CI] -1%, 21%, p=0.08) and radiologically-confirmed pneumonia (27%; [95% CI] 15%, 36%, p<0.0001) to our study's findings [5]. This difference may reflect the greater specificity of hypoxic pneumonia as an outcome, and thus provide a more accurate assessment of effect size. The outcome of hypoxic pneumonia was used in this study as it is a marker of severe pneumonia, is a precursor to mortality, and it is relatively easy to measure [32]. The WHO clinical pneumonia definition is sensitive but has low specificity for pneumococcal pneumonia. Studies have found the vaccine effectiveness of PCV against clinical pneumonia to be lower than for more specific pneumonia definitions, such as radiologically-confirmed pneumonia, and therefore larger samples are required to show any effect [22]. Additionally, we included pneumonia cases requiring oxygen supplementation in the outcome measure, as oxygen saturations were only recorded on admission. Therefore, using only “hypoxia” would not have captured the cases who deteriorated clinically during their hospitalisation.

This is the first study to describe this single-hospital approach to measure VE and provides a simple and feasible approach for other LMICs to adopt. Determining the effectiveness or impact of PCV in LMICs can be challenging. The quality of data and the consistency of clinician admission criteria is variable. For VE, case-control studies may be unreliable due to the lack of specificity of the pneumonia case definition and the choice of community controls may be biased. Drawing both cases and controls from patients that were hospitalised for the same condition, reduces bias that may otherwise occur from the association of vaccination and health seeking behaviour [16,33]. A previous study found that hospital controls had greater similarity of demographic characteristics to pneumonia cases than community controls [34]. We describe a variant of a case-control study design using hospital-based controls who had pneumonia but were not hypoxic. Although observational studies have inherent issues regarding confounding, we used PS analysis to adjust for this by balancing covariates between exposure groups. An advantage of the PS analysis is that it allows the balance of covariates to be reviewed (e.g. using standardised differences) [35,36]. Achieving covariate balance strengthens the internal validity of our study and gives greater confidence that any effect observed may be due to the causal relationship between PCV13 and protection from hypoxic pneumonia [35]. Furthermore, the PS model can be developed and checked before running the logistic regression for estimating VE and thus avoids results influencing methodology [37]. However, the validity of PS analyses also relies on a number of assumptions, including exchangeability (i.e. no unmeasured confounding), positivity (i.e. all children had a possibility of being vaccinated), and stable unit treatment assumption (i.e. the effect of vaccination for one individual does not depend on another person's vaccination status) [37]. Although we endeavoured to fulfil the assumptions, it is possible that some assumptions were not met. It is possible that some unmeasured confounders were not accounted for, which is a limitation of this study and all observational studies. However, our additional analysis of the control condition, which demonstrated PCV13 was not effective against total pneumococcal carriage, as expected, provides reassurance for our study design.

There were also a number of additional limitations to our study. Although all patients admitted with ARIs were eligible for the study, only patients who sought medical attention at Mahosot Hospital were included. This is likely to limit inclusion to middle-income families who live in the urban capital, which could affect generalisability of results to a wider population, whereby 66.9% live in rural areas and are poorer than urban dwellers [38]. Efforts were made to follow up and collect clinical information and PCV13 status for all children. In our study, PCV13 status was determined based on parent-held records, and if that was not available, vaccination was ascertained from health centre records where possible. Although these sources were preferred over parent recall, which has been found to be unreliable for determining immunisation status in other studies [39], it is still possible that misclassification of vaccination status occurred. Additionally, children with wheeze were included in the analysis. The WHO criteria for pneumonia has been shown to have poorer sensitivity and specificity in children with wheeze. However, both measures are improved with the addition of fever in the case definition [40]. Furthermore, the number of children in our study with wheeze was similar between groups. Finally, although this was a prospective study, undertaking research in LMICs can be challenging and missing data still occur even with a prospective design. To address the missing values we used multiple imputation and found a 35% PCV13 VE and a high level of evidence for likely effect, providing further support of the veracity of our findings.

In conclusion, these results show PCV13 is effective against hypoxic pneumonia in an Asian setting. Considering the high burden of pneumonia and limited access to treatment in LMIC settings, PCV13 is likely to substantially reduce childhood mortality in this region. These results provide a compelling argument to policy makers for the continued use of PCV13 in the childhood immunisation program and for its introduction into other similar countries. Moreover, we have described a low-cost and simple single hospital-based method for assessing PCV VE on pneumonia, which augments existing evaluation methods and advances implementation science methodology, especially for LMICs [41].

## Declaration of Competing Interest

KM, CDN and CS receive grants from Pfizer, outside the submitted work. The Institute of Research for Development (IRD) and Aix-Marseille University funds both ADP and the HRSV testing. PN receives grants from the Wellcome Trust. All other authors declare no competing interests. The opinion presented in this paper is that of the authors and does not reflect Pfizer. We have not been paid by a pharmaceutical company to write this article.

## Appendix Supplementary materials

Image, application 1
