# Supplementary material for: The effectiveness of the 13-valent pneumococcal conjugate vaccine against hypoxic pneumonia in children in Lao People's Democratic Republic: An observational hospital-based test-negative study
Source: Lancet Reg Health West Pac. 2020 Sep 6;2:100014. doi: 10.1016/j.lanwpc.2020.100014 (PMC8315332; doi:10.1016/j.lanwpc.2020.100014)
Supplement: Supplementary file 1 [file mmc1.docx]

**SUPPLEMENTARY MATERIALS**

**Supplementary Methods**

***Laboratory methods***

For pneumococcal testing, paediatric flocked swabs (Copan Diagnostics) were collected and stored according to World Health Organization guidelines.^1^ After collection, the swab was placed directly in a sterile cryovial of 1.0 ml skim milk, tryptone, glucose and glycerol (STGG) medium. The samples were stored at ultra-low temperature (i.e. -80°C)^1^, and then transported to the Murdoch Children’s Research Institute (Parkville, Australia) on dry ice for testing. DNA was extracted from 100 μl of STGG medium (following enzymatic treatment) using a MagNA Pure LC machine (Roche) using the DNA Isolation Kit III (Bacteria, Fungi) (Roche) as previously described.^2^ Sample DNA was screened by real-time quantitative polymerase chain reaction (qPCR) targeting the *lytA* gene, with the presence of pneumococci confirmed by DNA microarray.^2^

For HRSV testing, oral and nasal swabs were collected in viral transport medium (Sigma Virocult, MWE). 100 μL of both swabs were combined then extracted using the Cador Pathogen96 QIAcube HT kit (Qiagen) following the manufacturer’s instructions. Samples collected from December 2013 to December 2014 were submitted to the Fast-track Diagnostics respiratory pathogens 33 kit, multiplex probe based real-time PCRs for 33 pathogens. Samples collected after December 2014 were tested by probe based real-time RT-PCR using the iTaq™ Universal Probes One-Step reverse transcriptase kit (Bio-Rad) and primers/probe as previously described.^3^ A sample was considered positive for HRSV if the real-time RT-PCR assay had a CT value of <35.

***Multiple imputation***

Some children had unknown PCV13 vaccination status (144/826=17.4%) and unrecorded oxygen saturation levels (36/826=4.4%). There were also missing values in some of the variables in the propensity score model, namely: day care attendance (4/826=0.5%), HRSV (59/826=7.1%), maternal education (93/826=11.3%) and number of people in the household (2/896=0.2%). The missing values in these variables were imputed using multiple imputation by chain equations (MICE) using the *mi impute chained* command in Stata, with 40 imputed datasets created. Binary variables were imputed using logistic regression models, and continuous variables were imputed using linear regression models within the MICE procedure. The imputation model included hypoxic pneumonia status, PCV13 status and all variables in the propensity score model. As auxiliary variables, we included predictors of the incomplete variables (e.g. cough). The imputation procedure in Stata assumes that data are Missing at Random; to increase the likelihood of this assumption, we included variables in the imputation model that were predictive of missing data status (e.g. residence, ethnicity, date of enrolment). The unadjusted and PS analyses were performed separately on each imputed dataset, and the results combined using Rubin's rules.^4^

**References**

1. Satzke C, Turner P, Virolainen-Julkunen A, Adrian PV, Antonio M, Hare KM, et al. Standard method for detecting upper respiratory carriage of *Streptococcus pneumoniae*: updated recommendations from the World Health Organization Pneumococcal Carriage Working Group. *Vaccine* 2013; 32(1): 165-79.

2. Satzke C, Dunne EM, Choummanivong M, Ortika BD, Neal EFG, Pell CL, et al. Pneumococcal carriage in vaccine-eligible children and unvaccinated infants in Lao PDR two years following the introduction of the 13-valent pneumococcal conjugate vaccine. *Vaccine* 2019; 37: 296-305.

3. Fry AM, Chittaganpitch M, Baggett HC, Peret TC, Dare RK, Sawatwong P, et al. The burden of hospitalized lower respiratory tract infection due to respiratory syncytial virus in rural Thailand. *PLOS ONE* 2010; 5(11): e15098.

4. Rubin DB. Multiple imputation for nonresponse in surveys. New York: Wiley; 1987.

**Supplementary Table 1. Characteristics of hypoxic cases, by hypoxic on admission v non-hypoxic on admission but required supplementary oxygen during hospitalisation (n=285)**

|  | **Hypoxic on admission (n=139)** | **Non-hypoxic on admission but required supplementary oxygen (n=146)** | **p-value** |
| --- | --- | --- | --- |
| **Age in months, median (IQR)** | 7·7 (2·8-15·0) | 8·4 (2·8-16·6) | 0·86 |
| **Age, n (%)** |  |  |  |
| **<12 months** | 87 (62·6) | 96 (65·8) | 0·58 |
| **>12 months** | 52 (37·4) | 50 (34·2) |  |
| **Female, n (%)** | 60 (43·2) | 65 (44·5) | 0·82 |
| **Ethnicity (Lao Loum) n (%)** | 115 (82·7) | 123 (84·2) | 0·73 |
| **PCV13 status^1^, n/N (%)** |  |  |  |
| **Vaccinated** | 56/109 (51·4) | 59/120 (49·2) | 0·74 |
| **Undervaccinated** | 53/109 (48·6) | 61/120 (50·8) |  |
| **Comorbidities^2^, n (%)** | 28 (20·1) | 25 (17·1) | 0·51 |
| **HRSV positive, n/N (%)** | 44/132 (33·3) | 39/129 (30·2) | 0·59 |
| **Urban residence (Vientiane capital district)** | 97 (69·8) | 106 (72·6) | 0·60 |
| **Daycare attendance^3^, n/N (%)** | 8/138 (5·8) | 12/146 (8·2) | 0·43 |
| **Mother’s level of education higher than junior high school, n/N (%)** | 94/123 (76·4) | 89/127 (70·1) | 0·26 |
| **Season^4^, n (%)** |  |  |  |
| **Dry** | 90 (64·7) | 74 (50·7) | 0·016 |
| **Wet** | 49 (35·3) | 72 (49·3) |  |
| **Year of enrolment^5^, n (%)** |  |  |  |
| **2014** | 44 (31·7) | 32 (21·9) | 0·016 |
| **2015** | 30 (21·6) | 25 (17·1) |  |
| **2016** | 41 (29·5) | 42 (28·8) |  |
| **2017** | 20 (14·4) | 30 (20·5) |  |
| **2018** | 4 (2·9) | 17 (11·6) |  |
| **Number of other people in household, median (IQR)** | 5 (4-7) | 5 (3-6) | 0·058 |
| **Piped water source^6^, n (%)** | 65 (46·8) | 60 (41·1) | 0·34 |
| **Wood used for cooking^7^, n (%)** | 60 (43·2) | 82 (56·2) | 0·028 |
| **Assisted ventilation^8^, n/N (%)** | 13/138 (9·4) | 12/146 (8·2) | 0·72 |
| **ICU admission^9^ , n/N (%)** | 82/83 (98·8) | 73/76 (96·1) | 0·27 |
| **Death, n (%)** | 6 (4·3) | 3 (2·1) | 0.28 |

Legend: IQR: interquartile range; HRSV: human respiratory syncytial virus. For categorical variables, data are presented either as frequencies (n), or fractions (n/N) if there were missing data. ^1^Vaccinated: 0-11 months 2 doses of PCV13; >12 months 1 dose; undervaccinated: <11 months 0-1 dose; >12 months 0 doses of PCV13; unknown represents unreported or pending. ^2^Comorbidities included: concurrent infection, malnutrition, congenital heart disease, chronic lung disease, cancer, asthma, diabetes, prematurity or low birth weight. ^3^Attendance at daycare indicates children attended daycare for any length of time. ^4^Season: wet season refers to period between May to September, dry season refers to period between October to April. ^5^Year of enrolment refers to enrolment in study from 1^st^ of January to 31^st^ of December of that year (2013 was merged with 2014 due to low numbers), except 2017 which was January to June. ^6^Piped water source: Household had piped water supply. ^7^Wood used for cooking: Household had wood as primary cooking fuel as opposed to other sources. ^8^Assisted Ventilation: CPAP or mechanical ventilation. ^9^Intensive care unit (ICU) admission refers to admission for greater than 24 hours at any time during admission.

**Supplementary Table 2. Characteristics of children admitted with pneumonia, by known PCV13 vaccination status or unknown vaccination status (n=826)**

| **Characteristics** | **PCV13 status known**  **(n=682)** | **PCV13 status unknown (n=144)** | **p-value** |
| --- | --- | --- | --- |
| **Age in months, median (IQR)** | 11·5 (4·9-19·8) | 10·95 (4·1-20·1) | 0·98 |
| **Age, n (%)** |  |  |  |
| **<12 months** | 355 (52·1) | 75 (52·1) | 0·99 |
| **>12 months** | 327 (47·9) | 69 (47·9) |  |
| **Female, n (%)** | 290 (42·5) | 59 (41) | 0·73 |
| **Ethnicity (Lao Loum) n (%)** | 607 (89·0) | 110 (76·4) | <0·001 |
| **First recorded oxygen saturation^1^, n (%)** |  |  |  |
| **Hypoxic** | 109 (16) | 30 (20·8) | 0·2 |
| **Not hypoxic** | 543 (79·6) | 108 (75) |  |
| **Unknown** | 30 (4·4) | 6 (4·2) |  |
| **Supplementary oxygen used, n/N (%)** | 202/682 (29·6) | 54/143 (37·8) | 0·06 |
| **Comorbidities^2^, n (%)** | 82 (12·0) | 15 (10·4) | 0·6 |
| **HRSV positive, n/N (%)** | 213/637 (33·4) | 44/130 (33·8) | 0·93 |
| **Urban residence (Vientiane capital district)** | 566 (83·0) | 89 (61·8) | <0·001 |
| **Daycare attendance^3^, n/N (%)** | 85/679 (12·5) | 20/143 (14·0) | 0·63 |
| **Mother’s level of education higher than junior high school, n/N (%)** | 477/610 (78·2) | 87/123 (70  ·7) | 0·07 |
| **Season^4^, n (%)** |  |  |  |
| **Dry** | 369 (54·1) | 84 (58·3) | 0·35 |
| **Wet** | 313 (45·9) | 60 (41·7) |  |
| **Year of enrolment^5^, n (%)** |  |  |  |
| **2014** | 227 (33·3) | 27 (18·8) | <0·001 |
| **2015** | 167 (24·5) | 31 (21·5) |  |
| **2016** | 139 (20·4) | 46 (31·9) |  |
| **2017** | 110 (16·1) | 34 (23·6) |  |
| **2018** | 39 (5·7) | 6 (4·2) |  |
| **Number of other people in household, median (IQR)** | 5 (4-7) | 6 (4-7) | 0·02 |
| **Piped water source^6^, n (%)** | 373 (54·7) | 58 (40·3) | 0·002 |
| **Wood used for cooking^7^, n (%)** | 266 (39·0) | 79 (54·9) | <0·001 |
| **Assisted ventilation^8^, n/N (%)** | 17/681 (2·5) | 9 /143(6·3) | 0·02 |
| **ICU admission^9^, n (%)** | 136 (19·9) | 33 (22·9) | 0·7 |
| **Death, n (%)** | 5 (0·7) | 4 (2·8) | 0·9 |

Legend: IQR: interquartile range; HRSV: human respiratory syncytial virus. For categorical variables, data are presented either as frequencies (n), or fractions (n/N) if there were missing data. ^1^Oxygen saturation refers to first oxygen saturation measured either at or during admission: non-hypoxic: >90% O_2_ and hypoxic <90% O_2_. ^2^Comorbidities included: concurrent infection, malnutrition, congenital heart disease, chronic lung disease, cancer, asthma, diabetes, prematurity or low birth weight. ^3^Attendance at daycare indicates children attended daycare for any length of time. ^4^Season: wet season refers to period between May to September, dry season refers to period between October to April. ^5^Year of enrolment refers to enrolment in study from 1^st^ of January to 31^st^ of December of that year (2013 was merged with 2014 due to low numbers), except 2017 which was January to June. ^6^Piped water source: Household had piped water supply. ^7^Wood used for cooking: Household had wood as primary cooking fuel as opposed to other sources. ^8^Assisted Ventilation: CPAP or mechanical ventilation. ^9^Intensive care unit (ICU) admission refers to admission for greater than 24 hours at any time during admission.

**Supplementary Table 3. Characteristics of children admitted with pneumonia, by recorded oxygen saturation or unknown oxygen saturation status (n=826)**

| **Characteristics** | **Oxygen saturation status known**  **(n=790)** | **Oxygen saturation status unknown**  **(n=36)** | **p-value** |
| --- | --- | --- | --- |
| **Age in months, median (IQR)** | 11·2 (4·5-19·8) | 14·1 (7·3-20·6) | 0.14 |
| **Age, n (%)** |  |  |  |
| **<12 months** | 415 (52.5) | 15 (41·7) | 0·20 |
| **>12 months** | 375 (47.5) | 21 (58·3) |  |
| **Female, n (%)** | 335 (42·4) | 14 (38.9) | 0·68 |
| **Ethnicity (Lao Loum) n (%)** | 685 (86·7) | 32 (88·9) | 0·71 |
| **PCV13 status^1^, n (%)** |  |  |  |
| **Vaccinated** | 362 (45·8) | 15 (41·7) | 0·55 |
| **Undervaccinated** | 290 (36·7) | 15 (41·7) |  |
| **Unknown** | 138 (17.5) | 6 (16·7) |  |
| **Comorbidities^2^, n (%)** | 96 (12.2) | 1 (2.8) | 0·088 |
| **HRSV positive, n/N (%)** | 249/731 (34·1) | 8/36 (22·2) | 0·14 |
| **Urban residence (Vientiane capital district)** | 630 (79·7) | 25 (69·4) | 0·14 |
| **Daycare attendance^3^, n/N (%)** | 101/787 (12·8) | 4/35 (11·4) | 0·81 |
| **Mother’s level of education higher than junior high school, n/N (%)** | 537/697 (77·0) | 27/36 (75·0) | 0·78 |
| **Season^4^, n (%)** |  |  |  |
| **Dry** | 432 (54·7) | 21 (58·3) | 0·67 |
| **Wet** | 358 (45·3) | 15 (41·7) |  |
| **Year of enrolment^5^, n (%)** |  |  |  |
| **2014** | 238 (30·1) | 16 (44·4) | 0·002 |
| **2015** | 183 (23·2) | 15 (41·7) |  |
| **2016** | 182 (23·0) | 3 (8·3) |  |
| **2017** | 144 (18·2) | 0 (0) |  |
| **2018** | 43 (5·4) | 2 (5·6) |  |
| **Number of other people in household, median (IQR)** | 5 (4-7) | 5 (4-7) | 0·27 |
| **Piped water source^6^, n (%)** | 413 (52·3) | 18 (50) | 0·79 |
| **Wood used for cooking^7^, n (%)** | 333 (42·2) | 12 (33·3) | 0·29 |
| **ICU admission^8^, n (%)** | 166 (21·0) | 3 (8·3) | 0·7 |
| **Death, n (%)** | 9 (1.1) | 0 (0) | 0.52 |

Legend: IQR: interquartile range; HRSV: human respiratory syncytial virus. For categorical variables, data are presented either as frequencies (n), or fractions (n/N) if there were missing data. ^1^Vaccinated: 0-11 months 2 doses of PCV13; >12 months 1 dose; undervaccinated: <11 months 0-1 dose; >12 months 0 doses of PCV13; unknown represents unreported or pending. ^2^Comorbidities included: concurrent infection, malnutrition, congenital heart disease, chronic lung disease, cancer, asthma, diabetes, prematurity or low birth weight. ^3^Attendance at daycare indicates children attended daycare for any length of time. ^4^Season: wet season refers to period between May to September, dry season refers to period between October to April. ^5^Year of enrolment refers to enrolment in study from 1^st^ of January to 31^st^ of December of that year (2013 was merged with 2014 due to low numbers), except 2017 which was January to June. ^6^Piped water source: Household had piped water supply Attendance at daycare indicates children attended daycare for any length of time. ^7^ Wood used for cooking: Household had wood as primary cooking fuel as opposed to other sources. Piped water source: Household had piped water supply. ^8^Intensive care unit (ICU) admission refers to admission for greater than 24 hours at any time during admission.

**Supplementary Table 4. Characteristics of children admitted with pneumonia by PCV13 vaccination status and percentage standardised differences in covariates before and after inverse probability of treatment weighting. Standardised differences are shown for both complete case and multiple imputation analyses.**

|  | **Complete cases (n=570)** | | | **Complete case analyses (n=570)** | | **Multiple imputation**  **(n=826)** | |
| --- | --- | --- | --- | --- | --- | --- | --- |
| **Characteristics** | **PCV13**  **Undervaccinated (n=262)** | **PCV13**  **Vaccinated (n=308)** | **p-value** | **Standardised difference**  **Pre-IPTW** | **Standardised difference**  **Post-IPTW** | **Standardised difference**  **Pre-IPTW** | **Standardised difference**  **Post-IPTW** |
| **Age in months, median (IQR)** | 5.9 (2.0-21.5) | 12.9 (8.0-18.2) | <0.001 | 11·44 | -3·4 | 19·81 | 5·30 |
| **Sex, n (%)** | 114 (43.5%) | 119 (38.6%) | 0.24 | 9·92 | 4·46 | 3·22 | 3·39 |
| **Ethnicity (Lao Loum) , n(%)** | 216 (82.4%) | 292 (94.8%) | <0.001 | -39·69 | -6·82 | -47·78 | -4·66 |
| **Comorbidities^1^, n (%)** | 27 (10.3%) | 32 (10.4%) | 0.97 | -0·28 | 1·55 | -2·61 | 0·51 |
| **HRSV positive, n (%)** | 95 (36.3%) | 97 (31.5%) | 0.23 | 10·08 | 2·27 | 4·19 | 0·54 |
| **Urban residence (Vientiane capital district), n (%)** | 197 (75.2%) | 281 (91.2%) | <0.001 | 43·95 | 7·9 | 44·93 | 6·89 |
| **Attendance at daycare^2^, n (%)** | 33 (12.6%) | 37 (12.0%) | 0.83 | 1·77 | -3·76 | -10·02 | -3·63 |
| **Mother’s level of education greater than junior high school, n (%)** | 190 (72.5%) | 259 (84.1%) | <0.001 | -28·36 | -4·35 | -33·22 | -2·99 |
| **Season (wet)^3^, n (%)** | 136 (51.9%) | 129 (41.9%) | 0.017 | 20·19 | -·4 | 15·31 | 1·01 |
| **Year of enrolment^4^, n (%)** |  |  |  |  |  |  |  |
| **2014** | 133 (50.8%) | 73 (23.7%) | <0.001 | 71.5 | 5.83 | 65.03 | 4.73 |
| **2015** | 68 (26.0%) | 79 (25.6%) |  |  |  |  |  |
| **2016** | 48 (18.3%) | 86 (27.9%) |  |  |  |  |  |
| **2017** | 9 (3.4%) | 38 (12.3%) |  |  |  |  |  |
| **2018** | 4 (1.5%) | 32 (10.4%) |  |  |  |  |  |
| **Number of other people in household, median (IQR)** | 6 (4-7) | 5 (4-6.5) | 0.008 | -13·38 | 2·98 | -26·05 | -1·41 |
| **Piped water source, n (%)** | 112 (42.7%) | 195 (63.3%) | <0.001 | -42·11 | -3·99 | -40·89 | -2·75 |
| **Cooking fuel wood, n (%)** | 89 (34.0%) | 128 (41.6%) | 0.063 | -15·7 | -2·29 | -8·76 | -1·76 |

Legend: IQR: interquartile range; HRSV: human respiratory syncytial virus; IPTW: inverse probability of treatment weighting. ^1^Comorbidities includes: concurrent infection, malnutrition congenital heart disease, chronic lung disease, cancer, asthma, diabetes, prematurity or low birth weight. ^2^Attended care indicates children attended daycare for any length of time regularly. ^3^Season: wet season refers to period between May to September, dry season refers to period between October to April. ^4^Year of enrolment refers to enrolment in study from (2013 was combined with 2014 due to low numbers), except 2017 which was January to June. Summary statistics (n, %) are provided by year, but standardised differences in enrolment date were calculated in units of days.

**Supplementary Table 5. PCV13 vaccine effectiveness (VE) against total pneumococcal carriage (n=583)**

|  | **Unadjusted PCV13**  **VE (95% CI)** | **p-value** | **PCV13 VE**  **(PS^2^) (95% CI)** | **p-value** |
| --- | --- | --- | --- | --- |
| **Total Pneumococcal Carriage ^1^** | 10 (-26, 36) | 0·53 | -6 (-54, 28) | 0·78 |

Legend: ^1^Total Pneumococcal Carriage as recorded by nasopharyngeal swab. ^2^PS (Propensity score): individuals were weighted by the inverse probability of the conditional likelihood of being vaccinated using logistic regression on covariates: age, sex, season, day care attendance, number of adults in the household, maternal education, access to piped water, cooking fuel, ethnicity, date of enrolment, residing in a rural or urban setting, comorbidities and HRSV infection status.
